# Neutrophil‐to‐lymphocyte ratio in relation to trauma severity as prognosis factors in patients with multiple injuries complicated by multiple organ dysfunction syndrome: A retrospective analysis

**DOI:** 10.1002/iid3.1031

**Published:** 2023-09-26

**Authors:** Yong‐Ming He, Xing Liu, Si‐Yi Zhong, Qiu‐Hong Fu

**Affiliations:** ^1^ Department of Emergency Shenzhen Longhua District Center Hospital Shenzhen China; ^2^ Department of Emergency The Second People's Hospital of Futian District Shenzhen China; ^3^ Department of Public Health Guangdong Medical University Dongguan China

**Keywords:** injury severity score, multiple injuries, multiple organ dysfunction syndrome, neutrophil‐to‐lymphocyte ratio

## Abstract

**Objective:**

This study aimed to explore potential risk factors for the occurrence of multiple organ dysfunction syndrome (MODS) in patients with multiple injuries by evaluating neutrophil‐to‐lymphocyte ratio (NLR)‐associated trauma severity.

**Methods:**

This retrospective case–control study included 95 patients with multiple injuries, who were admitted to our hospital (between January 2018 and December 2020). Clinical data including gender, age, underlying disease, number of injury sites (NIS), injury severity score (ISS), hemoglobin level within 24 h of admission (HL‐24h), neutrophil count (NC), white blood cell count, platelet count (PC), NLR, d‐dimer level, activated partial thromboplastin time (APTT), complicated shock within 24 h of admission (CS‐24h), length of stay, as well as prognostic outcome was systematically analyzed. According to MODS occurrence, patients were divided into a MODS group (*n* = 27) and a non‐MODS group (*n* = 68). The risk factors affecting patients with multiple injuries complicated by MODS were identified using univariate and multivariate logistic regression analyses. Candidate risk factors were further analyzed using receiver operating characteristic (ROC) curves.

**Results:**

Univariate analysis revealed a significant difference between the MODS and non‐MODA groups in terms of NIS, ISS, HL‐24h, PC, APTT, d‐dimer level, CS‐24h, NLR, NC, prognostic outcome, and other indicators (*p* < .05). Multivariate logistic regression analysis showed that d‐dimer levels within 24 h of admission and ISS, NLR, and CS‐24h were significantly associated with multiple injuries complicated by MODS. Compared with the non‐MODS controls, the NLR in the MODS group showed a much higher level and tended to rise with the increase in ISS score, indicating a significant intergroup difference (*p* < .05). The ROC curve analysis results suggested that the NLR had good sensitivity and specificity for predicting the prognosis of patients with MODS with multiple injuries.

**Conclusion:**

d‐dimer level, ISS, NLR, and CS‐24h are important risk factors for MODS in patients with multiple injuries. Notably, NLR expression may be a good indicator of injury severity and predictor of the occurrence of MODS in patients with multiple injuries. Therefore, assessment of injury severity and coagulation function, active resuscitation, as well as prevention of infection should be emphasized during treatment of multiple injuries, to reduce and prevent the risk of MODS in patients with multiple injuries.

## 1 INTRODUCTION

Multiple injuries are serious injuries to more than two sites and organs caused by a single causative factor.^1^ Multiple injuries are severe, complicated, and prone to secondary infections. Within the first 12 h after trauma, the expression of some genes is upregulated in inflammatory cells and lymphocytes, leading to the overexpression of some inflammatory factors.^2^ If effective interventions are not provided in time after trauma, once an uncontrolled inflammatory reaction occurs, a chain waterfall reaction process of “systemic inflammatory response syndrome (SIRS) sepsis multiple organ dysfunction syndrome (MODS) multiple organ failure (MOF)” may develop gradually, eventually leading to patient death. The incidence of MODS after multiple injuries has been reported to be 8.3%–28%.^3^

MODS is a reversible dysfunction syndrome in which two or more organs suffer from severe trauma, infection, or other damages.^4^ MODS is the overactivation of systemic inflammation, which is a major cause of death in critically ill patients.^3^ However, owing to the reversibility of the process, early detection and attention to MODS and interruption of its progression by removing the cause and modulating inflammatory responses might be the key to treating MODS.

Neutrophil‐to‐lymphocyte ratio (NLR) is an emerging composite inflammatory biomarker that has been increasingly recognized as a rapid reflection of a patient's immune and inflammatory status.^5^ Compared with other inflammatory markers, such as C‐reactive protein, NLR has its own characteristics. The NLR integrates two distinct but complementary immune pathways, reflecting the degree of the body's stress response through lymphocyte counts. In addition, the neutrophil count (NC) reflects the degree of deterioration of the systemic inflammatory response. A lower ratio indicates a higher level of stress in the body.^6^

Currently, research on the application of NLR in multiple injuries is still rare. Therefore, we retrospectively reviewed the clinical data of patients with multiple injuries treated at our hospital between January 2018 and December 2021. Risk factors for MODS in patients with multiple injuries were systematically analyzed. Notably, the possibility of using the NLR to predict MODS occurrence in patients with multiple injuries was also explored in detail.

## 2 MATERIALS AND METHODS

### 2.1 General materials

Data from 95 patients with multiple injuries who were admitted to the emergency department of our hospital (between January 2018 and December 2020) were retrospectively analyzed. Of all patients, 75 were males, aged 11–74 years (average: 47.2 ± 4.56 years), and 20 were females, aged 9–67 years (average: 39.7 ± 3.74 years). According to the occurrence of MODS after injury, patients were divided into a MODS group (*n* = 39) and a non‐MODS group (*n* = 125), respectively. The MODS group consisted of 25 males and six females (average age: 45.3 ± 3.56 years). The non‐MODS group included 50 males and 14 females (average age: 46.3 ± 2.75 years). The injury types included 57 cases of car accident injury (49 males and eight females), 22 cases of fall from height injury (17 males and five females), and 12 cases of knife injury (nine males and seven females). The injury sites included 65 cases of cranial injury, 37 of chest injury, 24 of hepatic blunt trauma, 47 of spinal fractures, and 37 of pelvic fractures. Simultaneously, 50 healthy people who underwent physical examinations at our hospital health center were selected as the control group, according to the patients' basic information such as sex and age.

As shown in Table 1, the general information of the patients in the MODS and non‐MODS groups was not significantly different (*p* > .05).

**Table 1:** Comparisons of general information between the MODS and non‐MODS groups.

Items	MODS	Non‐MODS	*F*	*p*
Age (years)	38.14 ± 15.96	37.13 ± 12.79	1.774	.186
Gender ratio	25/6	50/14	3.532	.063

Abbreviation: MODS, multiple organ dysfunction syndrome.

### 2.2 Inclusion criteria

Inclusion criteria were as follows: (1) after examination, all diagnosis met the criteria of multiple injuries^1^; (2) patients with multiple injuries were scored according to The American concise injury grading standard (2005)^7^ with an injury severity score (ISS) score of more than 16 points (ISS ≥ 16); (3) survival time after injury should be more than 7 days (survival days > 7 days); (4) all patients were injured for the first time and remained healthy before illness; (5) all procedures were conformed to MODS diagnostic standard.^4^

### 2.3 Diagnostic criteria

Diagnostic criteria for multiple injuries^1^: two or more parts or organs are injured because of the same injury factor or at least one is fatal. ISS scoring standard^7^: the concise top‐level injury standard of the United States (2005) was used to score the severity of trauma in patients with multiple injuries. Diagnostic criteria for MODS^4^: (1) diagnostic criteria for cardiac dysfunction: (a) systolic blood pressure (<90 mmHg); (b) mean arterial pressure (<70 mmHg); and (c) severe arrhythmia such as shock, ventricular tachycardia, ventricular fibrillation, and myocardial infarction. A diagnosis can be made if one of three criteria (a, b, or c) is satisfied. (2) Diagnostic criteria for respiratory dysfunction: a diagnosis can be made if the oxygenation index (PaO_2_/FiO_2_) is <300 mmHg (PaO_2_/FiO_2_ < 300 mmHg). (3) Diagnosis of central nervous system dysfunction: (a) apathy, restlessness, lethargy, shallow, and deep coma; (b) Glasgow Coma Score ≤ 14; a diagnosis can be made if one of items (a and b) is available. (4) Diagnostic criteria for coagulation dysfunction: (a) platelet count (PC) < 100 × 10^9^/L; (b) prolonged or shortened activated partial thromboplastin time (APTT), coagulation time, and prothrombin time; 3P test was positive; a diagnosis could be made if one of items (a and b) was available. (5) Diagnosis of liver dysfunction: (a) total bilirubin > 20.5 μmol/L; (b) blood albumin < 28 g/L; a diagnosis can be made if one of items (a and b) is available. (6) Diagnostic criteria for renal dysfunction: (a) serum creatinine > 123.76 μmol/L; (b) urine volume < 500 mL/24 h; a diagnosis can be made if one of items (a and b) is available. (7) Diagnostic criteria for gastrointestinal dysfunction: (a) weakened or absent intestinal sounds; (b) positive hematemesis, black stool, and fecal occult blood test; a diagnosis can be made if one of the items (a and b) is available.

### 2.4 Exclusion criteria

Exclusion criteria were as follows: (1) no clear medical history; (2) the presence of chronic cardiopulmonary encephalopathy, liver disease, or tumor disease before the injury; (3) concomitant mental disorders; and (4) death occurring within 7 days after injury.

### 2.5 Study methods

The patients included in the study were divided into MODS and non‐MODS groups according to the MODS criteria. Univariate and multivariate analyses were subsequently performed on the following clinical indicators: sex, age, presence of underlying diseases, number of injury sites, ISS, hemoglobin level within 24 h of admission (HL‐24h), NC, white blood cell (WBC) count, PC, NLR, d‐dimer level, APTT, presence of complicated shock within 24 h of admission (CS‐24h), length of stay, and prognostic outcome. In addition, identified risk factors were further analyzed by receiver operating characteristic (ROC) curves to determine their diagnostic potential and the optimal threshold for predicting complicated MODS. The NLR levels between the different groups in the first 7 days after admission were also compared. Spearman's correlation analysis was conducted to determine the correlation between NLR and ISS scores.

### 2.6 Statistical analysis

Statistical software SPSS (ver. 17.0, USA) and the SPSSAU project (ver. 22.0) were used for the statistical analysis in this study. All measurement data are presented as mean ± SD using Student's *t* test with at least two independent samples. Non‐normally distributed measurement data are expressed as M (P25, P75) using a nonparametric test (Wilconxon rank‐sum test). Enumeration data were expressed as the number of cases using the chi‐square test. Binary logistic regression analysis was used for the multivariate analysis. ROC curve analysis was applied to discriminate risk factors for predicting complicated MODS in patients with multiple injuries, and the maximum value of the Youden index (sensitivity + 1 − specificity) was set as the best threshold. The diagnostic value was evaluated using the area under the curve (AUC). In general, diagnostic value was considered to be low (0.5 < AUC ≤ 0.7, moderate (0.7 < AUC ≤ 0.9), or high (AUC > 0.9). Correlation analysis was performed using the Spearman's rank test. The NLR value on Day 2 after admission in patients with multiple injuries was set as the *x* axis and the corresponding ISS score was set as the *y* axis. ROC curves were produced for NLR evaluation of prognosis and AUCs were calculated with 95% confidence interval (CI). Two‐sided values (*p* <  0.05) were considered significant.

## 3 RESULTS

### 3.1 Comparisons of NLR levels between healthy individuals and patients

**Figure 1:**
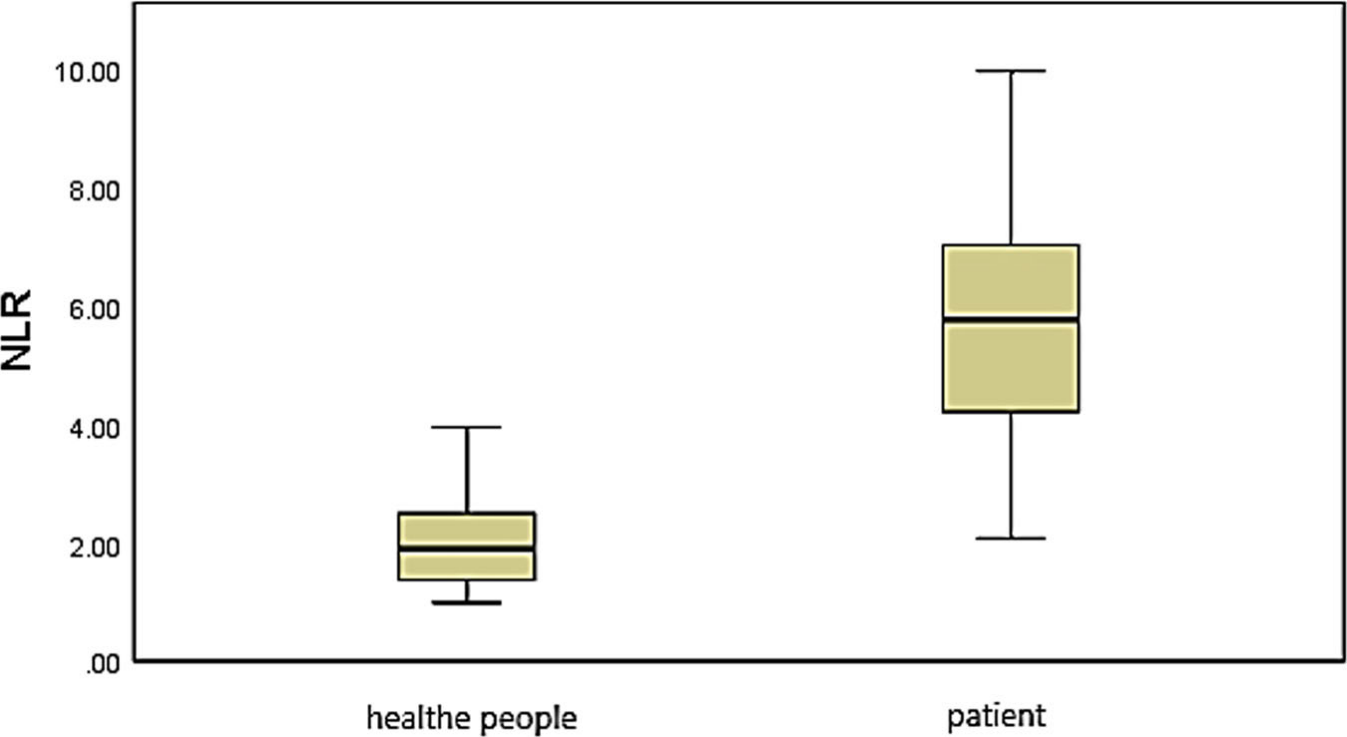
Comparisons of neutrophil‐to‐lymphocyte ratio (NLR) levels between healthy individuals and patients.

As illustrated in Figure 1, average NLR value was 5.55 ± 1.92 in patients, whereas for healthy individuals, average NLR value was 2.03 ± 0.76. There was a significant difference between the two groups. The results indicated that the healthy individuals had significantly lower NLR levels than the patients.

### 3.2 Univariate analysis of patients with multiple injuries complicated by MODS

In the univariate analysis, as shown in Table 2, there were statistically significant differences between the two groups in the number of injury sites, ISS, HL‐24h, PC, APTT, d‐dimer level, presence of CS‐24h, NLR, NC, prognostic outcome, and other indicators (*p* < .05). In contrast, no statistically significant differences were observed between the two groups in terms of sex, age, presence of underlying diseases, WBC count within 24 h of admission, length of hospital stay, or other indicators (*p* > .05).

**Table 2:** Univariate analysis of patients with multiple injuries complicated by MODS.

	Group (*x̄* ± *s*/%)				
Items	Non‐MODS (*n* = 64)	MODS (*n* = 31)	Total	*F*/*χ* ^2^	*p*
Injury site	2.53 ± 0.59	3.10 ± 0.70	/	16.950	.000**
ISS	17.34 ± 5.89	29.71 ± 8.02	/	72.248	.000**
HGB level within 24 h of admission	124.36 ± 18.26	138.65 ± 21.56	/	11.340	.001**
PLT	246.72 ± 89.94	308.19 ± 54.18	/	12.280	.001**
APTT	26.48 ± 9.49	38.95 ± 32.08	/	8.259	.005**
d‐Dimer	29.53 ± 10.43	39.74 ± 13.23	/	16.725	.000**
NLR	4.61 ± 1.41	7.68 ± 1.44	/	97.657	.000**
Neutrophil	9.38 ± 4.70	12.63 ± 7.14	/	7.041	.009**
Age	38.14 ± 15.96	37.13 ± 12.79	/	0.095	.759
WBC	13.59 ± 5.03	15.78 ± 8.10	/	2.612	.109
Hospital stay	27.27 ± 11.09	22.42 ± 14.35	/	3.276	.074
CS‐24h					
No	55 (85.94)	2 (6.45)	57 (60.00)	54.978	.000**
Yes	9 (14.06)	29 (93.55)	38 (40.00)		
Prognostic outcome					
Dead	1 (1.56)	18 (58.06)	19 (20.00)	41.670	.000**
Survival	63 (98.44)	13 (41.94)	76 (80.00)		
Gender					
Male	54 (84.38)	21 (67.74)	75 (78.95)	3.476	.062
Female	10 (15.63)	10 (32.26)	20 (21.05)		

Abbreviations: APTT, activated partial thromboplastin time; CS‐24h, complicated shock within 24 h of admission; HGB, hemoglobin; ISS, injury severity score; MODS, multiple organ dysfunction syndrome; NLR, neutrophil‐to‐lymphocyte ratio; PLT, platelet; WBC, white blood cell.

** *p* < .01.

### 3.3 Multivariate analysis of patients with multiple injuries complicated by MODS

According to the univariate analysis results, as shown in Table 3, indicators including the number of injury sites, ISS, HL‐24h, PC, APTT, d‐dimer level, presence of CS‐24h, NLR, NC, and prognostic outcome (*p* < .05) were included as independent variables in the multivariate logistic regression analysis, whereas the occurrence of MODS was included as a dependent variable. Logistic regression analysis results showed that the d‐dimer level within 24 h of admission, ISS score, NLR, and presence of CS‐24h were all significantly associated with multiple injuries complicated by MODS (*p* < .05).

**Table 3:** Multivariate analysis of patients with multiple injuries complicated by MODS.

Regression coefficient	Items	Regression coefficient		
	d‐Dimer	0.187* (1.965)		
	CS‐24h	4.346** (2.713)		
	ISS	0.314* (2.477)		
	NLR	0.910* (1.995)		
	Intercept	−22.982** (−3.157)		
	Likelihood ratio□	*χ* ^2^ (4) = 97.262, *p* = .000		
	Hosmer–Lemeshow test	*χ* ^2^ (8) = 3.653, *p* = .887		
Grouping regression model	Items	Total	Non‐MODS	MODS
	Constant	2.140** (5.545)	3.299** (6.269)	4.114** (5.754)
	ISS	0.163** (9.729)	0.076* (2.636)	0.121** (5.173)
	*n*	95	64	31
	*R* ^2^	0.504	0.101	0.480
	Adjustment *R* ^2^	0.499	0.086	0.462
	*F*	*F* (1,93) = 94.663, *p* = .000	*F* (1,62) = 6.949, *p* = .011	*F* (1,29) = 26.765, *p* = .000

Abbreviations: CS‐24h, complicated shock within 24 h of admission; ISS, injury severity score; MODS, multiple organ dysfunction syndrome; NLR, neutrophil‐to‐lymphocyte ratio.

* *p* < .05

** *p* < .01.

### 3.4 ROC curve analysis of ISS, d‐dimer, and NLR levels to predict complicated MODS in patients with multiple injuries

**Figure 2:**
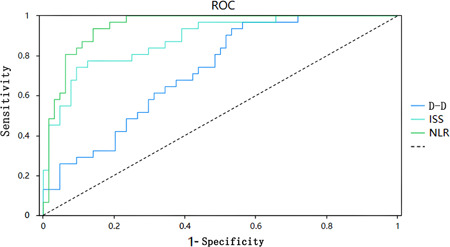
Receiver operating characteristic (ROC) curve analysis of injury severity score (ISS), d‐dimer (D‐D), neutrophil‐to‐lymphocyte ratio (NLR) levels to predict complicated multiple organ dysfunction syndrome in patients with multiple injuries.

ROC curve analysis was performed to analyze ISS, d‐dimer level, and NLR in patients with multiple injuries complicated by MODS. For ISS, the AUC of the ROC was AZ = 0.726 (95% CI: 0.667–0.784), which is considered a moderate diagnostic value for MODS prediction in patients with multiple injuries. For d‐dimer, the AUC of the ROC was AZ = 0.638 (95% CI: 0.571–0.706), which was evaluated as having low diagnostic value for MODS prediction in patients with multiple injuries. For the NLR, the AUC of the ROC was AZ = 0.735 (95% CI: 0.671–0.795), which was evaluated as a moderate diagnostic value for MODS prediction in patients with multiple injuries. As shown in Figure 2 and Table 4, the optimal threshold for ISS was 32 points, with a sensitivity of 68.1% and a specificity of 69.8%. The optimal threshold for d‐dimer was 21.84 mg/L with a sensitivity of 58.5% and a specificity of 66.3%. Multiple injuries complicated by MODS were set as predictive targets.

**Table 4:** ROC analysis of ISS, d‐dimer, and NLR levels to predict complicated MODS in patients with multiple injuries.

Indicator	AUC	SE	Significance	95% CI	
				Lower	Upper
ISS	0.726	0.030	0.000	0.667	0.784
d‐dimer	0.638	0.031	0.000	0.571	0.706
NLR	0.735	0.029	0.000	0.671	0.795

Abbreviations: 95% CI, 95% confidence interval; AUC, area under the curve; ISS, injury severity score; MODS, multiple organ dysfunction syndrome; NLR, neutrophil‐to‐lymphocyte ratio; ROC, receiver operating characteristic.

## 4 DISCUSSION

In terms of whether multiple injuries are associated with MODS, previous studies have proposed that in time correction of shock, ISS, acute physiological, and chronic health assessment II scores are independent risk factors for MODS predictions in patients with multiple injuries.^8^ In this retrospective study, a risk factor analysis of multiple clinical indicators was performed. The results showed that the independent risk factors affecting MODS in patients with multiple trauma included the number of injury sites, ISS, HL‐24h, PC, APTT, d‐dimer level, shock within 24 h of admission, NLR, NC, and prognosis.

An imbalance between the inflammatory and anti‐inflammatory responses is considered the underlying cause of MODS.^9^ Stress factors act on the body to activate inflammatory cells and release certain inflammatory mediators such as tumor necrosis factor‐α, interferon, interleukins IL‐1, IL‐2, IL‐6, and TXA2.^10^, ^11^ Although beneficial for bacterial killing, dysregulation of inflammatory and anti‐inflammatory responses also damage tissue cells, leading to SIRS. SIRS is mainly characterized by the excessive release of inflammatory mediators, suppressed monocyte/macrophage activity, reduced immune function, increased susceptibility to infection, and compensatory anti‐inflammatory response syndrome (CARS). The balance between the SIRS and CARS facilitates the maintenance of a stable internal environment. When SIRS predominates, the excessive activation and release of inflammatory cells induces shock, apoptosis, and MODS. In contrast, enhanced CARS can lead to dysfunction or failure of the immune system, eventually leading to MODS or MOF.^12^ In contrast, patients with multiple injuries have a high probability of infection for various reasons, including traumatic stress and immune system.^13^, ^14^ In this study, we found that infection was not the only independent risk factor for MODS in patients with multiple injuries, and that the risk of MODS in infected patients with multiple injuries was 25.13 times higher than that in patients without infection. Therefore, controlling and preventing infections and maintaining the balance of immune function are important for preventing MODS associated with multiple injuries.

NLR refers to the ratio of the absolute value of neutrophils to lymphocytes in the peripheral blood. Zahorec et al.^6^ first identified and proposed a correlation between NLR and prognosis in critically ill patients. When the body is under severe attacks, such as serious infection or excessive trauma, an increased neutrophilic and decreased lymphocytic response can be observed within 4–8 h and the NLR level increases as well. NLR, as a simple and readily available index for inflammation evaluation, can be calculated directly from the patient's neutrophil and lymphocyte counts without any other invasive examinations. Therefore, the NLR has been widely used to analyze the severity and poor prognosis of acute and chronic diseases such as pancreatitis, renal failure, coronary heart disease, and neoplasms.^15^, ^16^, ^17^, ^18^, ^19^, ^20^

In addition, the NLR has been applied to predict poor prognosis in patients undergoing abdominal surgery, severe trauma, and acute renal injury.^21^, ^22^, ^23^, ^24^ However, to date, there have been few reports on the application of the NLR in multiple injuries. Heffernan et al.^25^ found that neutropenia and lymphocytopenia were also present in trauma patients. Multiple injuries are characterized by rapid changes, high mortality, and susceptibility to infection. Moreover, multiple injuries induce the activation of the body's systemic inflammatory system, leading to the release of a large number of inflammatory factors, which in turn induce SIRS.^26^ During the acute phase of inflammation, many neutrophils are rapidly activated and migrate extensively throughout the whole body.^27^ Simultaneously, trauma and blood loss suppress the activity of T and B lymphocytes, thereby reducing the number of CD4+ T and natural killer cells and leading to low immunity, which greatly increases the probability of severe sepsis and multi‐organ system failure.^28^, ^29^, ^30^ Therefore, observed clinical reaction is described that during the early stages of multiple traumas, there is a significant increase in the number of neutrophils and a decrease in lymphocytes, accompanied by an increase in NLR levels. Neutrophils and lymphocytes play important roles in the immune response to infectious inflammation.^6^ In this retrospective study, we analyzed the reliability of NLR in evaluating the severity of multiple injuries and the relationship between NLR and poor prognosis. The results confirmed that NLR could be used as a simple biomarker to evaluate the severity of patients with multiple injuries and can accurately predict prognosis. A higher NLR indicated that patients with multiple injuries were more severely affected and had a poorer prognosis.

Traumatic shock is not only one of the major early complications in multiple trauma patients, but also one of the main causes of early death.^31^, ^32^ Immune cells are activated following shock in patients with multiple traumas. If a secondary infection is encountered, even if not severe, an uncontrolled excessive inflammatory response is induced, and cytokines and inflammatory mediators are released. Meanwhile, activation of the complement, coagulation, and fibrinolysis systems produces a waterfall effect, forming a vicious circle that eventually leads to MODS.^33^ In this study, total 89 patients with multiple injuries were complicated with shock, including 55 patients with traumatic shock in the MODS group, and 34 patients with traumatic shock in the non‐MODS group. The differences between the two groups were statistically significant. Therefore, shock is a major risk factor for MODS in patients with multiple injuries. Prompt and effective measures should be taken as early as possible to recover the shock status of patients with multiple injuries and maintain the effective circulating blood volume of the body to ensure the normal functional state of vital organs and tissues. This will not only improve the success rate of early treatment of patients with multiple injuries but also prevent further deterioration of the pathophysiological process and reduce the incidence of MODS.

The ISS is the most commonly used index for evaluating the severity of injury in patients with multiple injuries. Studies have shown that ISS can not only assess the severity of multiple injuries but also predict the prognosis of multiple injuries.^34^, ^35^ In this study, ISS was observed significantly much higher in MODS group than that detected from non‐MODS group (34.5 ± 7.2 vs. 23.3 ± 4.6, *p* < .05) Multivariate analysis indicated that ISS was an independent risk factor for MODS in patients with multiple injuries. In addition, ROC curve analysis showed that the ISS had moderate diagnostic value in predicting multiple injuries complicated by MODS.

Coagulation dysfunction plays an important role in MODS.^36^ Stress factors can release inflammatory mediators and activate the blood coagulation system, leaving blood in a hypercoagulable state. Thrombosis in the human microvasculature leads to impaired microcirculation, which in turn induces MODS. The d‐dimer is a fibrin degradation product produced by cross‐linking fibrin in the presence of fibrinolytic enzymes. Increased d‐dimer levels cause an increase in secondary fibrinolytic activity and can therefore be used as a marker to indicate hypercoagulability and fibrinolytic function in vivo.^37^ d‐dimer is not only associated with the severity of multiple injuries but also with prognosis.^38^, ^39^ The results of this study suggest that d‐dimer level is also a risk factor for multiple injuries complicated by MODS. However, the diagnostic value, sensitivity, and specificity of d‐dimer in predicting multiple injuries in MODS remain low.

During the treatment of multiple injuries, to prevent and reduce the risk of MODS complications in patients with multiple injuries, first, assess the condition of patients with multiple injuries comprehensively, especially ISS > 32 and d‐dimer levels > 21.83 mg/L within 24 h of admission; second, we should actively correct shock, supplement coagulation factors, correct coagulation disorders, as well as prevent and control infection.

## 5 CONCLUSION

Collectively, d‐dimer level, ISS, NLR, and CS‐24h are important risk factors for MODS in patients with multiple injuries. Notably, NLR expression may be a good indicator of injury severity and predictor of the occurrence of MODS in patients with multiple injuries. Therefore, assessment of injury severity and coagulation function, active resuscitation, as well as prevention of infection should be emphasized during treatment of multiple injuries, to reduce and prevent the risk of MODS in patients with multiple injuries. This study also has some limitations, such as its relatively small sample size, which may have statistical bias and requires a larger sample size for further confirmation.

## AUTHOR CONTRIBUTIONS

Qiu‐Hong Fu, Xing Liu, and Yong‐Ming He conceived the idea and conceptualized the study. Si‐Yi Zhong and Qiu‐Hong Fu collected the data. Yong‐Ming He and Si‐Yi Zhong analyzed the data. Xing Liu and Qiu‐Hong Fu drafted the manuscript, then Xing Liu and Si‐Yi Zhong reviewed the manuscript. All authors read and approved the final draft.

## CONFLICT OF INTEREST STATEMENT

The authors declare no conflict of interest.

## ETHICS STATEMENT

The study was conducted in accordance with the Declaration of Helsinki (as was revised in 2013). The study was approved by Ethics Committee of the Shenzhen Longhua District Center Hospital (No.2023‐045‐01). Written informed consent was obtained from all participants.

## Data Availability

The data sets used and/or analyzed during the current study available from the corresponding author on reasonable request.
